# SARS-CoV-2-free residual proteins mediated phenotypic and metabolic changes in peripheral blood monocytic-derived macrophages in support of viral pathogenesis

**DOI:** 10.1371/journal.pone.0280592

**Published:** 2023-01-19

**Authors:** Mohammad G. Mohammad, Naglaa S. Ashmawy, Ahmed M. Al-Rawi, Ameera Abu-Qiyas, Alshaimaa M. Hamoda, Rania Hamdy, Salam Dakalbab, Shahad Arikat, Dana Salahat, Mohamed Madkour, Sameh S. M. Soliman

**Affiliations:** 1 Department of Medical Laboratory Sciences, Collage of Health Sciences, University of Sharjah, Sharjah, UAE; 2 Research Institute for Medical and Health Sciences, University of Sharjah, Sharjah, UAE; 3 Department of Pharmacognosy, Faculty of Pharmacy, Ain Shams University, Abbassia, Cairo, Egypt; 4 College of Medicine, University of Sharjah, Sharjah, UAE; 5 Faculty of Pharmacy, Assiut University, Assiut, Egypt; 6 Faculty of Pharmacy, Zagazig University, Zagazig, Egypt; 7 College of Pharmacy, University of Sharjah, Sharjah, UAE; Children’s National Hospital, George Washington University, UNITED STATES

## Abstract

The large-scale dissemination of coronavirus disease-2019 (COVID-19) and its serious complications have pledged the scientific research communities to uncover the pathogenesis mechanisms of its etiologic agent, severe acute respiratory syndrome coronavirus-2 (SARS-CoV-2). Methods of unveiling such mechanisms are rooted in understanding the viral agent’s interactions with the immune system, including its ability to activate macrophages, due to their suggested role in prolonged inflammatory phases and adverse immune responses. The objective of this study is to test the effect of SARS-CoV-2-free proteins on the metabolic and immune responses of macrophages. We hypothesized that SARS-CoV-2 proteins shed during the infection cycle may dynamically induce metabolic and immunologic alterations with an inflammatory impact on the infected host cells. It is imperative to delineate such alterations in the context of macrophages to gain insight into the pathogenesis of these highly infectious viruses and their associated complications and thus, expedite the vaccine and drug therapy advent in combat of viral infections. Human monocyte-derived macrophages were treated with SARS-CoV-2-free proteins at different concentrations. The phenotypic and metabolic alterations in macrophages were investigated and the subsequent metabolic pathways were analyzed. The obtained results indicated that SARS-CoV-2-free proteins induced concentration-dependent alterations in the metabolic and phenotypic profiles of macrophages. Several metabolic pathways were enriched following treatment, including vitamin K, propanoate, and the Warburg effect. These results indicate significant adverse effects driven by residual viral proteins that may hence be considered determinants of viral pathogenesis. These findings provide important insight as to the impact of SARS-CoV-2-free residual proteins on the host cells and suggest a potential new method of management during the infection and prior to vaccination.

## Introduction

Severe acute respiratory syndrome coronavirus 2 (SARS-CoV-2), a positive sense single-stranded RNA virus, is the etiologic cause of the coronavirus disease 2019 (COVID-19) pandemic. As of February 9^th^, 2020, this highly contagious agent is responsible for an increasing pandemic trajectory [1] with its continuous appearance of elusive viral variants. To date, healthcare facilities struggle to ameliorate respiratory and immune complications induced by the virus, while only remdesivir and baricitinib are approved by the Food and Drug Administration (FDA). FDA has also authorized other therapies for emergency use against COVID-19, while their administration is governed by several age and patient condition-related restrictions [2].

Structurally, SARS-CoV-2 is composed of several proteins including the nucleocapsid protein (NP) and spike (S) protein. The S protein is functionally segregated into two parts designated as S1 and S2. Interactions between the S protein and the angiotensin-converting enzyme-2 (ACE-2) receptor, result in a cleavage event associated with the entry of the virus into the host cell, whereby S1 mediates the attachment, and S2 mediates the cell membrane fusion [3, 4]. Following entry, NP interacts with the viral RNA forming a complex that enhances viral transcription [5]. Although the proteins’ structural role is clear, their functional role as free residual proteins requires further investigation. Severe COVID-19 cases are often associated with compromised immune responses [6, 7]. Macrophages are important mediators of the inflammatory immune responses [8]. They are phenotypically and functionally categorized into different groups; M1 (pro-inflammatory) and M2 (anti-inflammatory) macrophages are among the extensively studied subsets [9]. Microbial agents often alter the macrophages’ intrinsic profile, creating an environment that enhances their chances of survival and dissemination [10]. For instance, virus-mediated alterations of a host cell’s metabolism cater to the virus’ needs in accelerating viral genome replication and providing substrates for virion production [11]. This invasion mediates yet another intrinsic change in the host in the form of an immune response. An antiviral immune response is conferred by the proinflammatory subset of macrophages, which are suggested to play a role in prolonged inflammatory phases and adverse immune responses, including excessive cytokine secretions [12, 13]. Therefore, analyzing the metabolic profiles that instigate such immune responses in the macrophages is essential for understanding the viral pathogenesis.

The effect of SARS-CoV-2 residual proteins on the metabolism of macrophages is not fully understood. Several studies have highlighted the significance of residual and secretory peptides on metabolic reprograming in macrophages, as a response to abnormal homeostasis and functional plasticity [14, 15]. It is important to describe such metabolic alterations in macrophages, to gain insight as to the pathogenesis of SARS-CoV-2, and its associated complications; thus aiding progression and improvements in antiviral therapies and vaccination protocols.

## Materials and methods

### Viral proteins

SARS-CoV-2 nucleocapsid protein (NP), spike 1 (S1), spike receptor binding domain (RBD, ECD) proteins, and ACE-2 Fc chimera human protein were purchased from GenScript (NJ, USA). Proteins were reconstituted as per manufacturer’s instructions and aliquots of the stock solutions were stored at -80°C until use.

### Preparation of peripheral blood monocyte-derived macrophages (PBMMs)

Enrichment of PBMMs was performed following a previously published protocol [16]. Blood collection was approved by the University of Sharjah ethics committee (REC-19-07-19-01) and healthy donors signed a written consent form. Blood samples were EDTA treated and pooled in 50 ml falcon tubes. To separate the peripheral blood mononuclear cells (PBMCs), 12.5 ml pooled blood was over-layered onto 10 ml Histopaque-1077 (Sigma, St. Louis, MO, USA), followed by centrifugation at 400 x g for 20 min at room temperature, with brakes turned off. PBMCs in the interface were aspirated and washed one time with warm PBS. The pellets were suspended in 1 ml RPMI media and viable cells were counted using Trypan blue vital dye. For the proliferation assay, the cells were seeded into 96-well tissue culture plates at a density of 5x10^4^ cells/200 μL of RPMI-1640 media supplemented with 10% fetal bovine serum (FBS) (Sigma, USA) and 1% Penicillin/Streptomycin (Sigma, USA). For metabolomics and flow cytometry, cells were seeded into T-25 flasks (Corning, USA) at a density of 5x10^6^ cells/5 ml RPMI-1640 media, supplemented as previously mentioned. Flasks were incubated for 24h at 37°C and 5% CO_2_. Floating non-monocytic cells were isolated by gentle removal of the supernatants, followed by a gentle wash with pre-warmed PBS. The cells were then treated with the aforementioned proteins diluted in RPMI-1640.

### Proliferation assay

To study the PBMMs proliferation in response to SARS-CoV-2 and ACE-2 proteins, a WST proliferation assay (Cat # 15092, Intron Biotechnology, South Korea) was conducted. This allowed for the detection of mitochondrial activity through pinpointing the number changes in PBMMs viability. WST was applied to the PBMMs according to the manufacturer’s instructions. In brief, the obtained PBMMs were incubated with NP, ECD, S1, and ACE-2 proteins at 1, 10, 100, and 1000 ng/mL. Lipopolysaccharide (LPS) and Interleukin 4 (IL4) were added as positive controls at 100 [17], and 15 ng/mL [18], respectively. Each treatment was conducted in 6 replicas, representing 3 independent experiments. PBMMs were then incubated with the proteins for 8 days, followed by incubation with WST for 4h. Readings were performed using a spectrophotometer (BioTek ELx808, CA, USA) and according to the manufacturer’s instructions.

### Morphological observation of macrophages

Macrophages were isolated using the previously mentioned PBMMs extraction method. The cells were then subsequently treated with SARS-CoV-2 proteins, including NP (100 and 1000 ng/mL), ECD (100 ng/mL), S1 (1 and 100 ng/mL), as well as chimera human protein ACE-2 (1000 ng/mL). Untreated cells were employed as a negative control, while LPS (100 ng/mL) and IL-4 (15 ng/mL) served as positive controls. After 8 days, the cells were observed under an IX73 inverted light microscope (Olympus, Japan) at 10x objective lens. Random image sets were captured using the Olympus cell Sens entry software. Spindle- and round-shaped macrophages were quantified using the ImageJ software. The counts were then normalized to depict the number of spindle- and round-shaped cells per unit area.

### Macrophage staining for flow cytometry

PBMMs were cultured and treated with the aforementioned proteins. PBMMs were then scraped and transferred to 15 mL falcon tubes, followed by centrifugation at 250 x g for 10 min at 4˚C. The supernatants were discarded, and the pellets were then resuspended in 1 mL staining washing buffer (SWB), containing 1% FBS and 1 μM EDTA in PBS. The cell counts were determined using Trypan blue vital stain at 1:2 final dilution, using Neubauer improved hemocytometer. One million cells were stained for 25 min, on ice, in the dark with CD14 (BD, United States) and CD16 (R&D, United States). The samples were then washed one time and the pellets were resuspended in 250 μL SWB. Results were acquired using BD FACS Aria III (BD, United States) and then analyzed using FlowJo V10 flow cytometry analysis software (BD, United States).

### Extraction and preparation of samples for metabolomics analysis

The extraction and preparation of secreted metabolites, following the macrophage treatment with the aforementioned proteins, were performed according to Soliman *et al*, 2020 [19]. Supernatants from treated PBMMs were collected, centrifuged at 4000 x g to remove any debris, and stored at -80°C until use. The filtered supernatants were extracted with ethyl acetate and the solvent was evaporated using a rotatory evaporator. The metabolite extracts were derivatized by a mixture of *N*-trimethylsilyl-*N*-methyl trifluoroacetamide and trimethylchlorosilane (MSTFA + 1% TMS), according to previously published protocol [20].

### Gas chromatography-mass spectrometry (GC-MS) analysis

GC-MS-QP 2010 Ultra System (Shimadzu, Kyoto, Japan) integrated with LabSolutions GC-MS software (v1.20) was employed for the metabolomics analysis. A Restek Rtx®−5ms column (30.0 m ×0.25 mm, 0.25 μm) was utilized for metabolites’ separation. Helium (99.9%) was the carrier gas, at a flow rate of 1.0 mL/min. The oven temperature was initially set to 60°C for 2 min, then raised to 310°C by 50°C/min and kept at this temperature throughout the analysis. The interface auxiliary and ionization temperatures were set at 250°C. Metabolites were analyzed in full scan mode, in the range of 50–650 amu. Moreover, a total volume of 10 μL was injected in splitless mode by employing AOC-20i Auto Injector (Shimadzu, Kyoto, Japan). Finally, GC total ion chromatograms (TIC) and fragmentation patterns of all metabolites were identified using NIST/EPA/NIH Mass Spectral Library (NIST 14), with a run time of 43.67 min per sample.

### Multivariate analysis

The GC-MS mass signals were collected and manually filtered to eliminate any unidentified ions. Signals belonging to the same ion category were grouped together; the data were then normalized according to the total peak height. The table with metabolic data from all replicas was generated (triplicate per each extract) and assessed. Principal component analysis (PCA) and partial least squares-discriminate analysis (PLS-DA) were carried out to visualize the grouping tendencies within the samples. Potential biomarkers were extracted from the values of a variable importance plot (VIP). Furthermore, analysis of the heat maps and hierarchical clustering was conducted to discover probable grouping and patterns in the metabolic coverage. All chemometric statistical analysis was carried out using MetaboAnalyst 5.0 (http://www.metaboanalyst.ca/) [21] and according to Alaa et al., 2021 [22].

### Metabolites’ set enrichment analysis (MSEA)

Metabolomics coverage between the different samples was conducted using MSEA. The enriched metabolic pathways were examined, using over-representation analysis, in comparison to the total annotated metabolites in the same pathway. False Discovery Rate was calculated based on the multiple testing correction statistical method [23] and MetaboAnalyst 5.0 (http://www.metaboanalyst.ca/) was utilized to conduct the statistical analysis [24]. Potential biomarkers were further subjected to pathway analysis using the KEGG analysis (http://www.kegg.jp/kegg/pathway.html) metabolic pathways database, in order to identify the related metabolic pathways [25].

### Statistical analysis

GraphPad Prism software (San Diego, California, USA) was used to analyze data, and the results were expressed as the average ± standard error of the mean (SEM). One-way analysis of variance (ANOVA) was used to compare quantitative differences between groups in each of the data sets of the proliferation assay, flow cytometric analysis, and morphological counts. *P*-value < 0.05 represented significant statistical difference.

## Results

### SARS-CoV-2-free proteins showed concentration-dependent alterations in the proliferation and phenotypic subsets of human macrophages (PBMMs)

**Fig 1:**
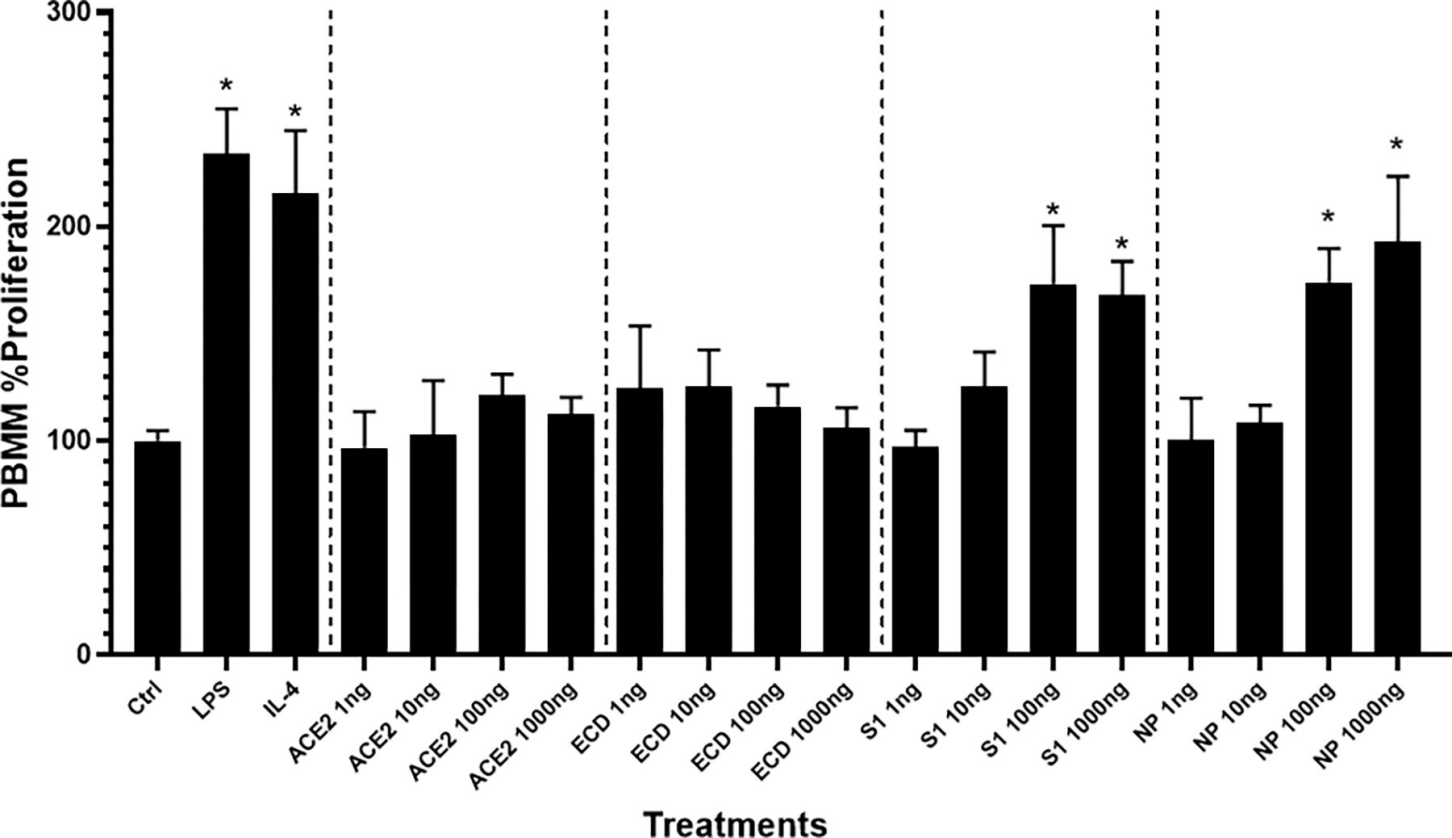
Proliferation of PBMMs in response to SARS-CoV-2 proteins and human ACE-2. WST was used to evaluate PBMMs percentage proliferation, as calculated in comparison to untreated cells. The average ± SEM of 4 independent experiments with 6 replicas is shown. The results were analyzed using one-way ANOVA. * *P*< 0.0001 considered as significantly different as compared to untreated controls.

**Fig 2:**
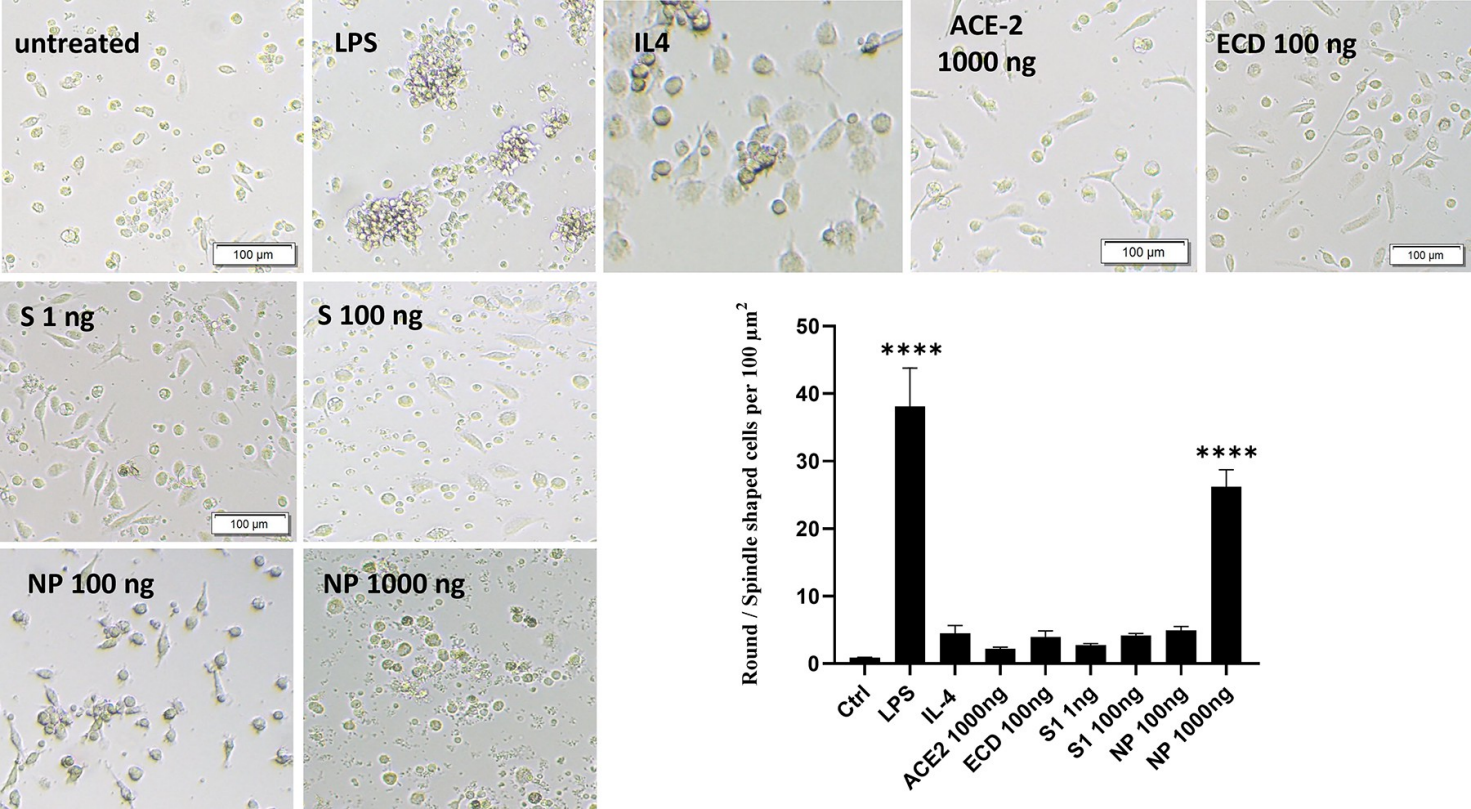
Morphological changes in PBMMs in response to SARS-CoV-2 proteins and human ACE-2. (**A**) Cells treated with SARS-CoV-2 proteins and human ACE-2 protein. Untreated cells served as the negative control, while LPS and IL-4 were positive controls. Following the end of their treatments, 10–15 random photos of the cultures were imaged by Olympus microscope magnification 10x, Scale bar = 100 μm. (**B**) Counts of round- and spindle-shaped PBMMs per 100 μm^2^, represented as a ratio of round- to spindle-shaped cells. The results were then analysed using one-way ANOVA and presented as mean ± SEM of three independent experiments. **** *P*< 0.0001 considered as significantly different from untreated control.

S1 and NP at concentrations of 100 and 1000 ng/mL, respectively, significantly (*P* < 0.0001) induced macrophage proliferation compared to untreated cells (**Fig 1**). However, ECD and ACE-2 did not show significant effects at increased concentrations (**Fig 1**). Macrophages treated with SARS-CoV-2 proteins were morphologically and phenotypically assessed. Morphological changes, in correlation to activated macrophages, have been previously reported as an indicator of the macrophage’s functional subset [26]. Consequentially, the presence of spindle- and round-shaped macrophages indicate the presence of pro-inflammatory and anti-inflammatory responses, respectively. PBMMs treated with ACE-2 and S1 (1 ng/mL) showed the lowest ratio of round-to-spindle shaped cells, while NP-treated cells showed the highest ratio (*P* < 0.0001), similar to that of LPS-treated cells (**Fig 2**). This indicates a macrophage shift towards an M1 pro-inflammatory state mediated by NP, or to an M2 anti-inflammatory state mediated by ACE-2 and S1.

**Fig 3:**
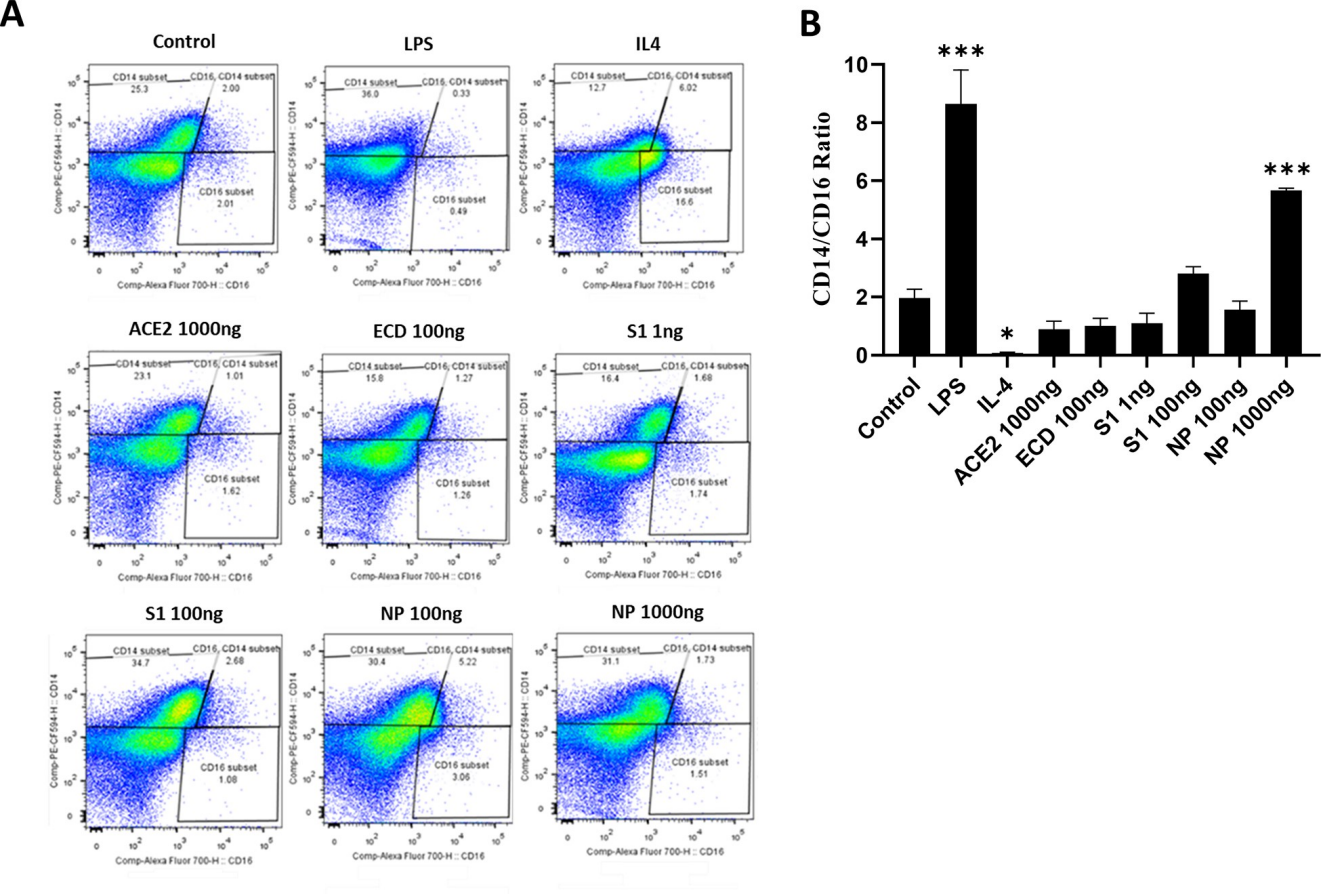
Phenotypic changes of PBMMs in response to SARS-CoV-2 proteins and human ACE-2 treatment. IL4 and LPS were employed as controls. (**A**) Phenotypic profiling of macrophages, following treatment with SARS-CoV-2 and human ACE-2 proteins, was examined by assessing the differential expression of the two main macrophage markers CD14 and CD16. (**B**) Data were represented as a ratio of CD14 to CD16 positive cells. The results were analysed using one-way ANOVA and presented as mean ± SEM. * *P*< 0.05, *** *P*< 0.001, and **** *P*< 0.0001, which are significantly different from the untreated controls.

The phenotypic changes of PBMMs, in response to treatment with SARS-CoV-2 proteins, were assessed using CD14 and CD16 surface markers. PBMMs treated with SARS-CoV-2 proteins showed upregulated CD14, similar to that of LPS-treated cells (**Fig 3A**). S1- and NP-treated PBMMs showed concentration-dependent CD14 expression. Compared to the untreated control, S1 at 100 ng/ mL (*P*<0.05) and NP at 1000 ng/ mL (*P*<0.0001) caused a significant increase in the CD14/CD16 ratio, similar to LPS (*P*<0.0001) (**Fig 3B**). Both the morphological and phenotypic observations indicate an inflammatory PBMM response to treatment with NP and S1 proteins.

### SARS-CoV-2-free proteins induced significant concentration-dependent metabolic changes to the macrophages

Following treatment of macrophages with the viral proteins (S1, ECD, and NP) and human ACE-2, metabolomics analysis of extracted supernatants was performed using GC-MS, in comparison to LPS and IL-4 as positive controls and vehicle as a negative control. A total of 26 major metabolites were selected and subjected to comparative analysis using multivariate statistical analysis, partial least-squares discrimination analysis (PLS-DA) and hierarchical clustering analysis (HCA). The discrimination of metabolites was performed using VIP scores of the PLS-DA model.

**Fig 4:**
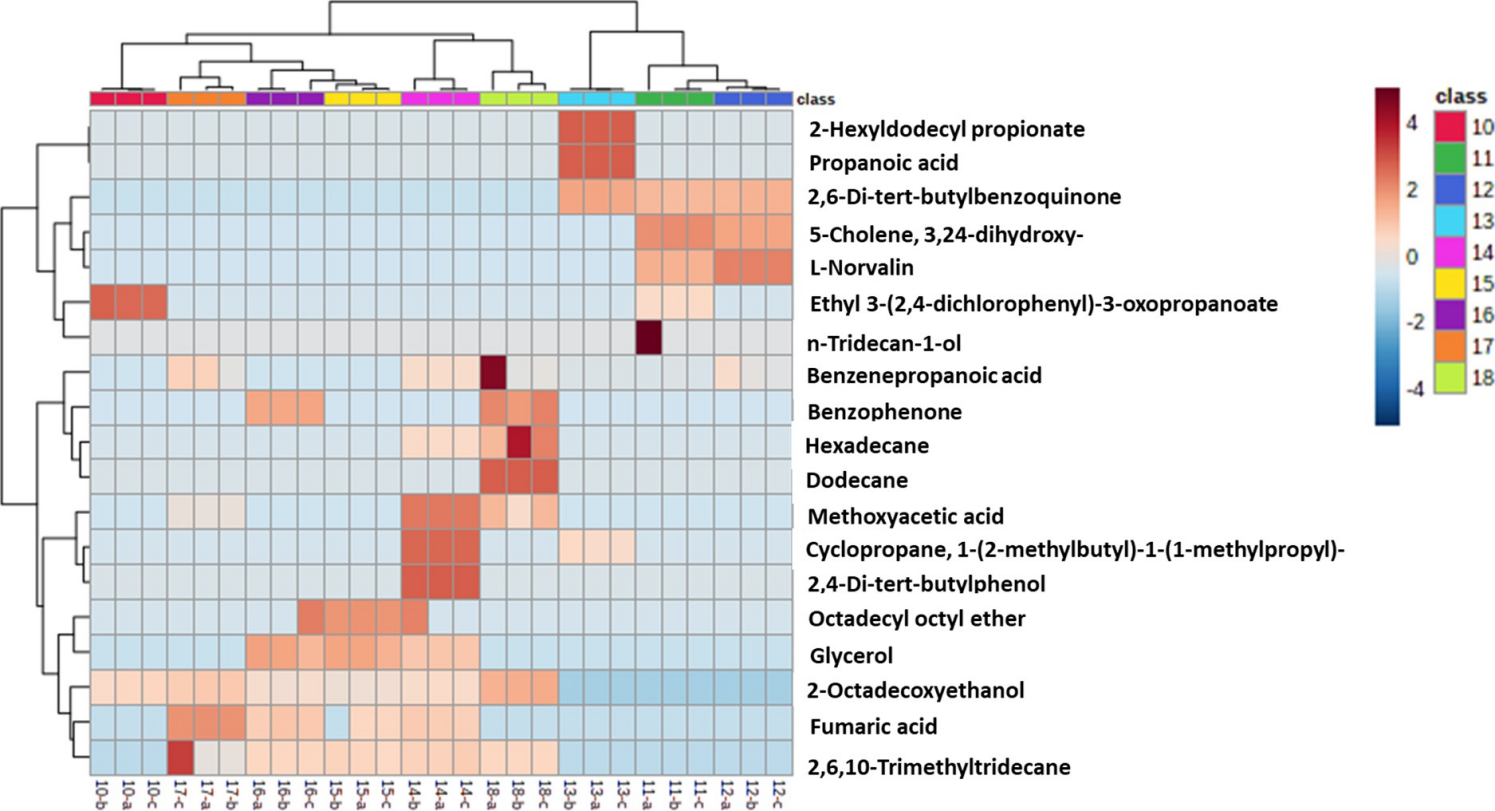
Unsupervised hierarchical clustering and heatmap of the identified metabolites in the extracted samples (rows) through different supernatants (columns).

**Fig 5:**
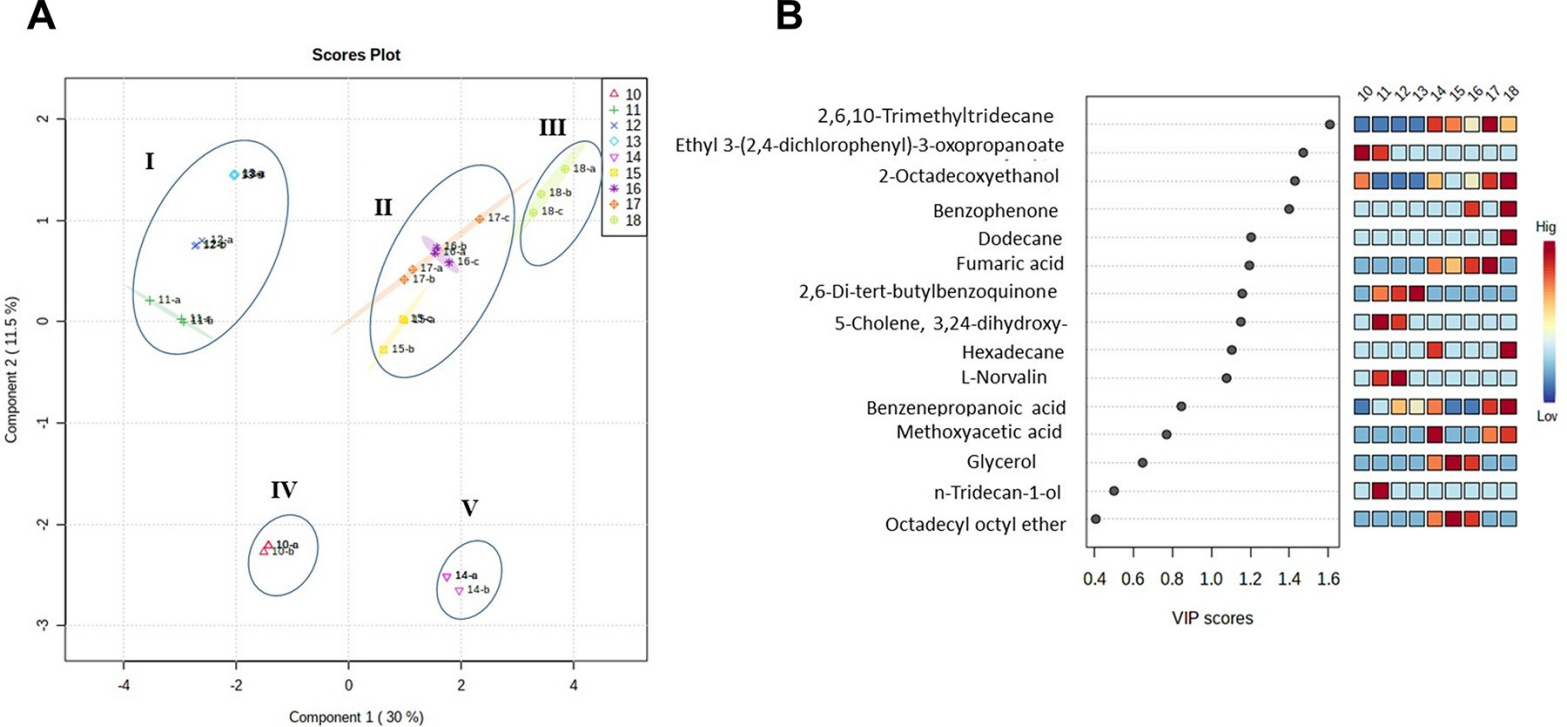
PLS-DA model for biomarker identification and selection. (**A**) 2D score plot and (**B**) VIP score plot of GC-MS data of treated samples versus controls.

HCA, shown in **Fig 4**, was carried out to show the clustering patterns of metabolites released in response to the aforementioned viral proteins and human ACE-2, compared to that of the controls. The data indicated a significant difference in the metabolites released as a result of each treatment. PLS-DA, in relation to the effect of NP at 1000 ng/ mL, NP at 100 ng/ mL, ACE-2, ECD, S1 at 100 ng/ mL and S1 at 1ng/ mL, displayed five main metabolite clusters compared to controls. PLS-DA score plot indicated that the metabolic changes due to S1 at a lower concentration (sample #17), S1 at a higher concentration (sample #16), and ECD (sample #15) were clustered in one group (group #II). While metabolite changes due to NP at a higher concentration (1000 ng) (sample #10) were separately clustered in group #IV away from those at a lower concentration (100 ng) (sample #11) in another cluster (group #I) (**Fig 5A**). Similarly, LPS (sample #13) and IL-4 (sample #12) were clustered in group #I, indicating a distinct effect on macrophage metabolism (**Fig 5A**). ACE-2 (sample #14) was separately clustered in group #V (**Fig 5A**). These results indicate a variable pattern of activation in response to different types and concentrations/doses of viral proteins. On the other hand, metabolites extracted from the negative control (untreated cells, sample #18) were present in one group (group #III), fully separated from all other treatments (**Fig 5A**). This finding suggests that a significant effect is modulated by viral proteins and ACE-2 on the metabolic behavior of macrophages. Moreover, the PLS-DA model revealed that 10 metabolites showed VIP score values greater than one **(Fig 5B)**.

**Fig 6:**
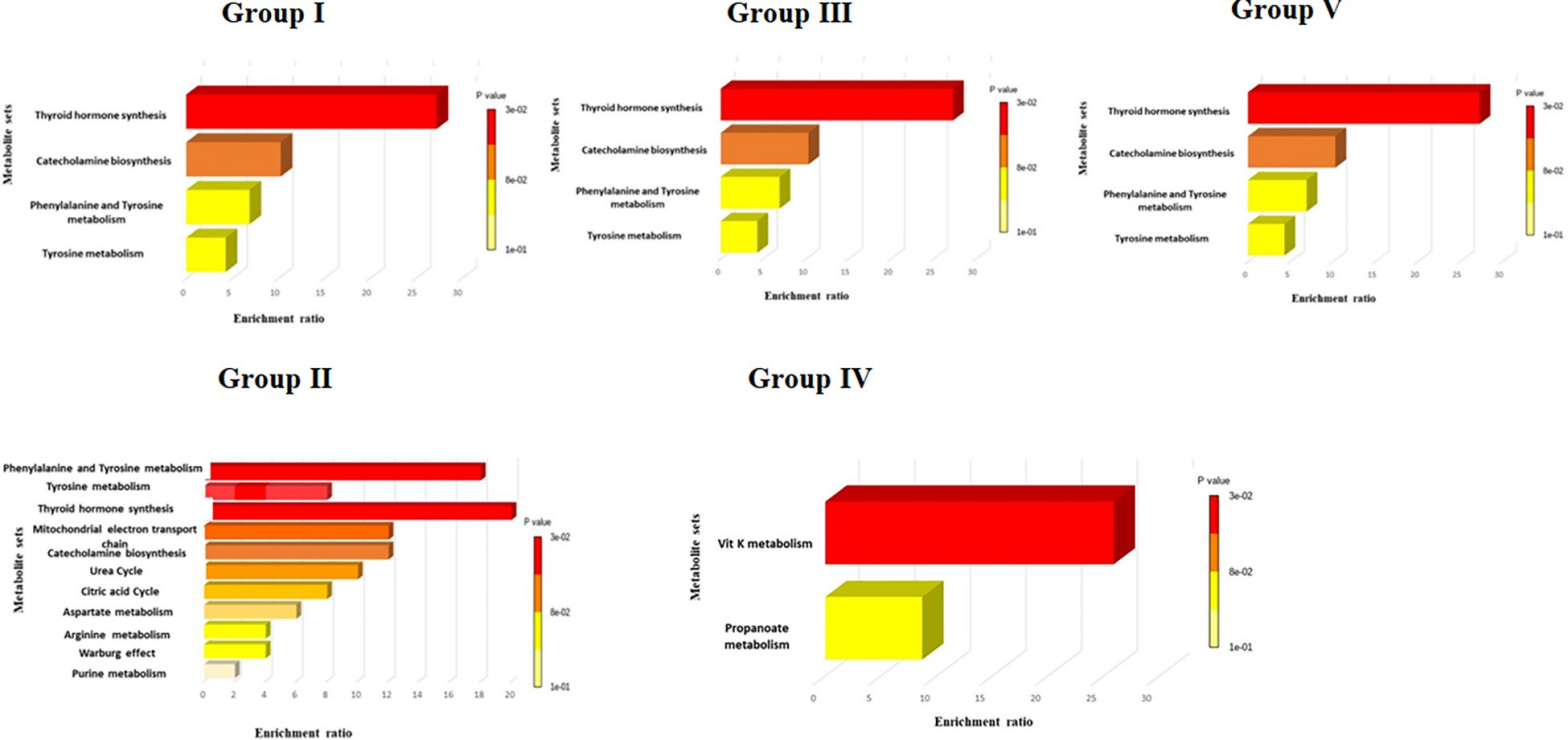
Diagram showing enriched metabolic pathways for each metabolic group.

Metabolites set enrichment analysis (MSEA) was further conducted to determine the metabolic pathways that were enriched following protein treatment (**Fig 6**). MSEA showed that ECD and S1 treatments (group #II metabolites) enriched the metabolic pathways related to a vigorous inflammatory response. It also depicted high energy generation and consumption, including the activation of urea cycle, aspartate metabolism, D-arginine and D-ornithine metabolism, galactose metabolism, mitochondrial electron transport chain and arginine and proline metabolism (**Fig 6**). In contrast, NP showed concentration-dependent metabolic enrichment. At low concentrations, NP enhanced the metabolic pathways in a manner similar to those observed in LPS and IL4 treatments, resulting in anti-inflammatory or proinflammatory responses (group #I). Interestingly, at the high concentration, NP resulted in a significant upregulation of vitamin K and propanoate metabolism (group #IV, **Fig 6**). In addition, S1 and NP treatment enhanced the metabolic pathways related to thyroid hormone synthesis.

## Discussion

**Fig 7:**
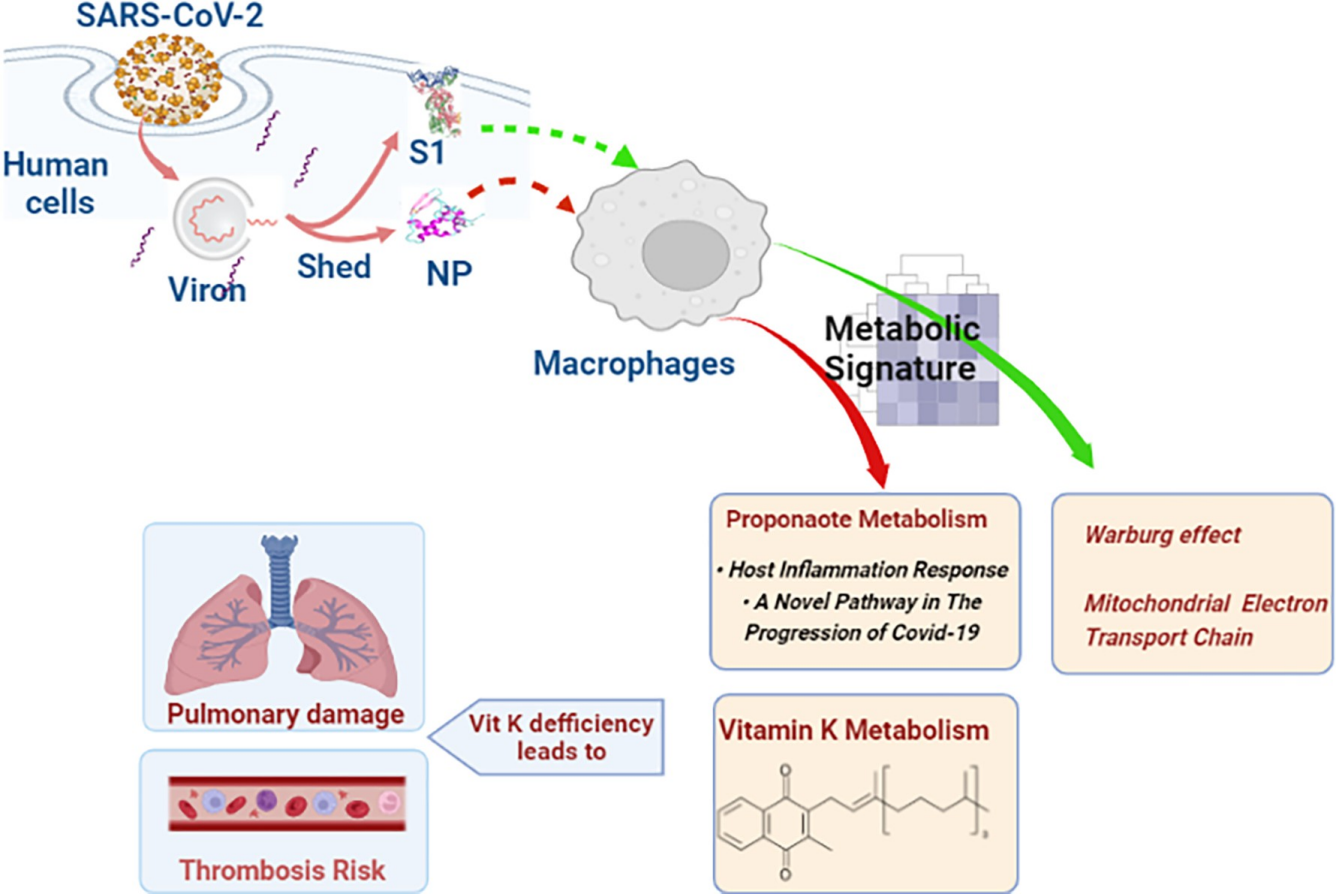
Model illustrating the effect of SARS-CoV-2-free proteins on macrophages.

Previous studies demonstrated the important influence of a macrophage’s metabolic status on the fate of the virus, inducing either the eradication or persistence of the infection [27]. In our study, we observed the effects of free SARS-CoV-2 residual proteins on macrophages’ metabolism, as illustrated with the model summarized in **Fig 7**. The virions of SARS-CoV-2 interact with the target human cells via the viral S protein and host ACE-2 receptor. This interaction is followed by the cleavage of S protein to S1 and S2, which facilitates the internalization of the virion into the host cells [28, 29]. However, the pertinent effects of the free residual S proteins, resulting from this cleavage, remain ambiguous. Similarly, the effects of NP after shedding are largely unknown. SARS-CoV-2 virions have not yet been detected in the blood of infected subjects. However, the viral proteins released during the cleavage event, the virion assembly in host cells, or from the damaged cells at the site of infection, may be detected in the circulation [30, 31]. The effects mediated by these proteins during SARS-CoV-2 infection require further investigation. One proposed consequence of residual SARS-CoV-2 proteins is the downregulation of ACE-2 activity, which would result in modulating the functions of angiotensin II, the indigenous substrate for ACE-2, resulting in inflammatory outcomes and pathological coagulability [29]. SARS-CoV-2 was previously demonstrated to antagonize the STAT1 antiviral response pathway [32–34]. NP of SARS-CoV-2 was recognized for its role in suppressing the STAT1 phosphorylation [35]. Suppression of STAT1 pathway leads to compensatory STAT3 hyper-activation, which is associated with elevating plasminogen activator inhibitor-1 (PAI-1), and consequently leads to affliction with coagulopathies [36].

Our results illustrate a significant enrichment in vitamin K metabolism in response to NP treatment, which may complement the previous work on the potential induction of coagulopathies and the thromboembolism events present in severe COVID-19 cases [37]. Vitamin K also plays a role in the activation of matrix Gla protein (MGP), which protects against vascular and pulmonary elastic fiber damage [38]. Recently, an inverse correlation between the levels of inactive vitamin K dependent matrix Gla protein (dp-ucMGP) and vitamin K status was detected, which is significantly elevated in COVD-19 cases in comparison to healthy controls [39]. In COVID-19, vitamin K insufficiency is linked to cytokine storms and the severity of pulmonary diseases [40]. The severity of COVID-19 pneumonia was linked to the depletion of vitamin K [41], therefore, the administration of vitamin K as adjuvant therapy would play an important role in the prevention and treatment of severe COVID-19 cases [40]. The emerging role of vitamin K administration as adjuvant therapy showed clinical outcomes in hospitalized COVID-19 patients [42]. Furthermore, propanoate metabolism was altered following NP treatment. This is consistent with the alteration effects of SARS-CoV-2 on the gut microbiota and the production of short-chain fatty acids (SCFAs), which play an important role in regulating the production of antimicrobial peptides and in activating the mucosal immune system [43]. Propanoate metabolism is positively associated with lipogenesis and host inflammation responses. A recent study identified the association between propanoate metabolism and progression of COVID-19 [44]. Exploiting the variations in fatty acid metabolism, which may be easily regulated by dietary modification, SCFA in the diet has been encouraged as a valuable strategy for the production of anti-inflammatory cytokines [45]. Dietary supplements with sodium propanoate have proven to be effective in lowering the intensity of viral inflammatory immune responses, and could potentially reduce the severity of COVID-19 infections [46].

Inhibition of glycolysis has been known to affect macrophage phagocytosis [47]. Certain viruses such as noroviruses, can optimally live in normal glycolytic conditions of macrophages, through Akt activation, which slows down apoptosis and prolongs the viral replication [48]. Studies on monocytes and macrophages in HIV-infected patients show differential glycolysis effects; metabolites involved in macrophage glycolysis were reduced, in addition to lipid alterations [49]. The main source of energy for these infected cells was glutamine, glutamate, and α-ketoglutarate, while their inhibition can cause significant elimination of the infected macrophages [49]. Another study showed upregulated glucose uptake in monocytes following HIV infection, which led to reduced inflammatory cell apoptosis [50, 51]. In SARS-CoV-2-infected patients, glycolytic activity is upregulated in inflammatory macrophages, which is associated with a significant increase in cytokine production and viral replication [52]. Similarly, as a result of S1 protein treatment, Warburg effect was enhanced, which is a known outcome of SARS-CoV-2 infection [53]. Furthermore, treatment of macrophages by S1 protein caused enrichment of metabolic pathways related to mitochondrial electron transport chain. This virus may also evolve to modulate immunometabolism and mitochondrial function, to ensure its replication at the cost of the host’s cellular energy [54]. Both S1 and NP enriched thyroid hormone synthesis, which is consistent with the reported effect of SARS-CoV-2 infection in the development of thyroid dysfunction [55].

Our results demonstrated that S1 and NP provoked significant macrophage proliferation, in a dose-dependent manner. This is in accordance with COVID-19 disease severity due to viral loads [56, 57]. Interestingly, we have detected that SARS-CoV-2-free proteins can cause similar adverse effects. The resulting free S1 protein, following S protein cleavage [58–60], has been speculated to play a role in the pathogenesis of COVID-19 disease [29] such as the induction of endothelial injury [61, 62] and cytokine production in PMNC [63]. Another study reported that the recombinant proteins of SARS-CoV-2 NP and S2-ECD, but not S1 subunit or RBD domain of S protein, can remarkably induce the pro-inflammatory cytokines/chemokines in treated human primary PBMCs and monocyte-derived macrophages [64]. Furthermore, Letarov *et al*. hypothesized that virions in the extracellular matrix may result in a significant release of S1 subunits, due to cell surface protease TMPRSS2-mediated cleavage, prior to the virions entry into the host cells [29]. These products are predicted to saturate both the soluble and host cells-attached ACE-2 receptors, resulting in virions neutralization and preventing further virion attachment to the host cells. Collectively, these findings strongly indicate that S1-ECD may induce anti-inflammatory responses or have no direct effect on the proliferation of macrophages, while S2-ECD functions as a pro-inflammatory mediator. Additionally, previous studies confirmed that S1 subunit is responsible for receptor recognition, in which the RBDs play a role in the stabilization of the pre-fusion state of S2 subunit that mediates the membrane fusion process [60, 65].

Treatment of macrophages with SARS-CoV-2 proteins leads to M1 classical macrophage polarization, mostly due to higher concentrations of S1 or NP. Morphological studies showed that treatment with NP 1000 ng/ml induces cell rounding, resembling that of LPS-treated cells, in turn alluding to a pro-inflammatory phenotype [66]. These findings were further confirmed by flow cytometry. Findings also indicated that an increase in NP or S1 concentrations, leads to a proportional increase in the number of classical CD14+ monocytes, which induces pro-inflammatory conditions. Additionally, as previously reported, patients with a high viral load at the early stage of the disease can develop a hyperactive immune response due to the respective increase in the classical CD14+ pro-inflammatory macrophages [67]. These macrophages induce dysregulation and increase cytokine production, leading to the development of a cytokine storm that in turn exacerbates patient complications [68]. Therefore, it can be suggested that the use of NP-specific antibodies may neutralize the pathogenicity and associated immunological complications, thus halting disease progression.

## Conclusion

In summary, SARS-CoV-2 proteins induced immunological and metabolic changes in PBMMs. Our findings illustrated an increased tendency in the proliferation and proinflammatory phenotypic changes of macrophages, in a dose-dependent manner, as a response to SARS-CoV-2 S1 and NP treatments. These changes were associated with distinct metabolomic changes that exacerbated the inflammatory responses. Moreover, the evident increase in vitamin K and propanoate metabolism in response to NP treatment or the Warburg effect and the mitochondrial electron transport chain in response to S1 protein, may suggest a potential contribution to complications experienced by COVID-19 patients. The inflammatory role of PBMMs, driven by metabolic alterations resulting from residual viral proteins, should be considered when developing therapeutic strategies against SARS-CoV-2 infection.

## Abbreviations

ACE-2: Angiotensin-converting enzyme-2
EDTA: Ethylene diamine tetra-acetic acid
FBS: Fetal bovine serum
FDA: Food and Drug Administration
GC-MS: Gas chromatography-mass spectrometry
HCA: Hierarchical clustering analysis
IL-4: Interleukin 4
KEGG: Kyoto encyclopedia of genes and genomes
LPS: Lipopolysaccharide
MDMs: Monocyte-derived macrophages
MGP: Matrix Gla protein
MSEA: Metabolite set enrichment analysis
MSTFA: *N*-Trimethylsilyl-*N*-methyl trifluoroacetamide and trimethylchlorosilane
NP: Nucleocapsid
PAI-1: Plasminogen activator inhibitor-1
PBMMs: Peripheral blood-derived monocytic macrophages
PBNC: Peripheral blood mononuclear cells
PBS: Phosphate-buffered saline
PCA: Principal component analysis
PLS-DA: Partial least squares-discriminate analysis
RBD: Spike receptor-binding domain
RPMI: Roswell Park Memorial Institute Medium
S: Spike
SARS-CoV-2: Severe acute respiratory syndrome coronavirus 2
SCFAs: Short-chain fatty acids
SEM: Standard error of the mean
SWB: Staining washing buffer
TMPRSS2: Human transmembrane serine protease-2
VIP: Variable importance plot
WST: Water-soluble tetrazolium salt
